# Dark triad personality traits are associated with decreased grey matter volumes in ‘social brain’ structures

**DOI:** 10.3389/fpsyg.2023.1326946

**Published:** 2024-01-12

**Authors:** Artem Myznikov, Alexander Korotkov, Maya Zheltyakova, Vladimir Kiselev, Ruslan Masharipov, Kirill Bursov, Orazmurad Yagmurov, Mikhail Votinov, Denis Cherednichenko, Michael Didur, Maxim Kireev

**Affiliations:** ^1^Russian Academy of Science, N.P. Bechtereva Institute of Human Brain, Saint Petersburg, Russia; ^2^Department of Psychiatry, Psychotherapy and Psychosomatics, Medical Faculty, RWTH Aachen University, Aachen, Germany; ^3^Saint Petersburg State University, Saint Petersburg, Russia

**Keywords:** machiavellianism, psychopathy, narcissism, K-means, vbm, emotional regulation, empathy, reward system

## Abstract

**Introduction:**

Personality traits and the degree of their prominence determine various aspects of social interactions. Some of the most socially relevant traits constitute the Dark Triad – narcissism, psychopathy, and Machiavellianism – associated with antisocial behaviour, disregard for moral norms, and a tendency to manipulation. Sufficient data point at the existence of Dark Triad ‘profiles’ distinguished by trait prominence. Currently, neuroimaging studies have mainly concentrated on the neuroanatomy of individual dark traits, while the Dark Triad profile structure has been mostly overlooked.

**Methods:**

We performed a clustering analysis of the Dirty Dozen Dark Triad questionnaire scores of 129 healthy subjects using the k-means method. The variance ratio criterion (VRC) was used to determine the optimal number of clusters for the current data. The two-sample t-test within the framework of voxel-based morphometry (VBM) was performed to test the hypothesised differences in grey matter volume (GMV) for the obtained groups.

**Results:**

Clustering analysis revealed 2 groups of subjects, both with low-to-mid and mid-to-high levels of Dark Triad traits prominence. A further VBM analysis of these groups showed that a higher level of Dark Triad traits may manifest itself in decreased grey matter volumes in the areas related to emotional regulation (the dorsolateral prefrontal cortex, the cingulate cortex), as well as those included in the reward system (the ventral striatum, the orbitofrontal cortex).

**Discussion:**

The obtained results shed light on the neurobiological basis underlying social interactions associated with the Dark Triad and its profiles.

## 1 Introduction

The ‘social brain’ is one of the brain basis model of social interactions that incorporates many elements of various systems involved in socially significant aspects of human behavior (Adolphs, 2009). According to recent studies, the ‘social brain’ model comprises components of the reward system (Bhanji and Delgado, 2014), the Theory of Mind (ToM) neural system and its various domains, affective and cognitive, as well as the systems supporting empathy (Maliske and Kanske, 2022). However, despite considerable research efforts directed at the regularities of the structural and functional organization of such systems, their neurobiological foundations remain uncertain.

Hence, a major goal of neurophysiological research should be to investigate how these brain systems are engaged in human social behavior. Such research on the neurobiological basis of human social behavior should take into account the influence of social intelligence or other personality characteristics (such as the Dark Triad) that might be associated with both the characteristics of human social activity and their neurobiological basis (Gundogdu et al., 2017; Segel-Karpas and Lachman, 2018; Back, 2021). We assume that such framework has already proved to be fruitful in studies that address the brain foundations of social behavior. For example, in recent morphometric and fMRI studies, we have found a link between gray matter morphometric characteristics of the basal ganglia (caudate nuclei) and its functional connectivity with the ToM neuronal system components, and social intelligence characteristics (Myznikov et al., 2021; Votinov et al., 2021). Using the Guilford-Sullivan test (O’Sullivan and Guilford, 1976), we reported higher gray matter volumes in the caudate nuclei of subjects with high social intelligence scores. Moreover, the level of social intelligence positively correlated with the degree of functional connectivity between the head of the right caudate nucleus and the brain areas associated with the ToM network, i.e., the right temporoparietal junction and the precuneus.

From the perspective of personality psychology, altogether with social intelligence, social behavior is affected by a set of non-pathological personality traits (non-pathological personalities), first described in theory of the Dark Triad by Paulhus and Williams (2002). The triad includes: (1) narcissism – characterized by excessive self-esteem and arrogance (Levy et al., 2011); (2) psychopathy – characterized by a lack of empathy and remorse, as well as such phenotypic domains as disinhibition, arrogance, and courage (Patrick, 2022); (3) Machiavellianism – characterized by a tendency to manipulate and use others for manipulator’s purposes (Jones and Paulhus, 2009). According to a number of researchers, the combination of these traits constitutes the Dark Core of personality conceived as the Dark Factor of Personality, or the D-factor (Moshagen et al., 2018). Moshagen et al. (2018) defines the D-factor as “the tendency to maximize one’s individual utility — disregarding, accepting, or malevolently provoking disutility for others —accompanied by beliefs that serve as justifications.” Dark traits are considered as specific manifestations of a general, basic dispositional behavioral tendency that in fact manifests the Dark Factor of Personality. It is believed that individuals with more prominent dark personality traits tend to be more prone to antisocial and dangerous behavior, which cannot but affect the nature of social interactions (Lämmle and Ziegler, 2014; Sijtsema et al., 2019; Pechorro et al., 2022). Accordingly, the meta-analysis by Muris et al. (2017) demonstrated the connection between the dark traits and a number of unfavorable psychosocial factors. Namely, the level of narcissism was associated with difficulties in interpersonal relationships, while Machiavellianism – with difficulties in interpersonal relationships and antisocial tactics. The largest number of adverse psychosocial factors was associated with psychopathy and included aggression, socio-emotional deficit, sexual behavior disorders, and antisocial tactics.

Based on the literature, the sum of the test results (D-factor) is widely used to characterize dark personality traits. There are even well-founded recommendations to use composite scores rather than subscale scores for one of the dark triad tests because subscales contain small amounts of reliable variance beyond the general factor (Persson et al., 2019). At the same time, as some studies show, the Dark Triad construct is more complex than a mere sum of traits – rather a function of their multifaceted interaction, which poses the main problem of psychometric measurement concerning the Dark Triad (Kam and Zhou, 2016; Trahair et al., 2020; Truhan et al., 2021). The literature discusses whether the Dark Triad traits are independent behavioral predictors or whether they should be combined within the framework of an integral assessment of the complex of traits (the dark core of personality). In favor of this view, a recent study by McLarnon and colleagues used exploratory bifactor modeling by structural equations (B-ESEM) of the results of the SD3 questionnaire (McLarnon, 2022). This approach helped identify latent profiles of the triad in a healthy population based on four factors (narcissism, Machiavellianism, psychopathy, and the general D-factor). The profiles are defined as follows: (1) troublemakers (high levels of narcissism, psychopathy, and D-factor, a low level of Machiavellianism); (2) self-absorbed (low levels of Machiavellianism, psychopathy, and D-factor, a high level of narcissism); (3) manipulators (high levels of Machiavellianism and D-factor, low levels of psychopathy and narcissism); (4) exploiters (high levels of psychopathy and Machiavellianism, low levels of narcissism and D-factor). In another study, using the structural classification method, three categories were identified: benevolent (low machiavellianism, psychopathy and narcissism), intermediate malevolent (medium machiavellianism, psychopathy and narcissism) and high malevolent (high machiavellianism, psychopathy and narcissism; see Garcia and MacDonald, 2017). The multifaceted structure of the triad has also been explored in other studies, some of which use another comprehensive questionnaire – the Dirty Dozen Dark Triad (DDDT, Garcia and Rosenberg, 2016; Maneiro et al., 2020).

Despite the heterogeneity of the results obtained through the integrative approach that simultaneously considers all the subtests of the Dark Triad questionnaire, recent studies validate its efficiency. In this regard, we need to emphasize that we have not been able to find separate neurobiological studies that account for such integrity of the Dark Triad subtests indicators. As of now, the most widespread in the field are studies of the neuroanatomic organization of individual Dark Triad traits based on specialized psychometric questionnaires. Few studies have shown a positive correlation between the level of Machiavellianism (measured by a separate expanded specialized psychometric test MACH-IV) and the volume of some structures, such as subcortical nuclei, the prefrontal cortex, and the insula (Verbeke et al., 2011; Gong et al., 2023). Likewise, the meta-analysis by De Brito et al. demonstrated a gray matter volume decrease in the medial orbitofrontal and dorsolateral prefrontal cortex in psychopathy (De Brito et al., 2021). Additionally, a number of studies have shown a positive correlation between the level of psychopathy (assessed using PCL-R questionnaires) and the volume of subcortical nuclei (Korponay et al., 2017; Lam et al., 2017), in particular, the putamen, caudate nuclei, although an inverse relationship is also reported (Vieira et al., 2015). Meanwhile, the level of narcissism assessed using an Neuropsychiatric Inventory questionnaire (NPI) positively correlated with the volume of a number of structures, including the medial and the ventromedial prefrontal cortex, the dorsolateral prefrontal and orbitofrontal cortex, the middle anterior cingulate cortex, and the insula (Nenadić et al., 2021). Notably, in the case of the pathological variant – the narcissistic personality disorder – the degree of manifestation of the disease negatively correlated with the volume of the medial prefrontal and dorsolateral cortex (Nenadic et al., 2015). Summarizing the above studies, we should witness a clear lack of research on the neurobiological foundations underlying the general construct of the Dark Triad rather than its individual features.

As regards the neuroimaging studies that applied voxel morphometry, we would like to draw attention to at least two major weaknesses that we attempt to address in the present study. First, as mentioned, these cited studies consider individual Dark Triad traits within the framework of specialized tests overlooking the general construct (an integrative evaluation of all traits). Second, these studies tend to disregard various Dark Triad profiles. In view of the above, our work was designed to conduct a morphometric study of dark personality traits, taking heed of the integral characteristics of the Dark Triad. To this end, with the present study we tried to overcome the main, in our opinion, shortcomings of the extant literature by using the general Dark Triad questionnaire, DDDT, and the k-means data clustering algorithm that allows an integral assessment of the Dark Triad traits. This would allow us to adequately approach the multifactorial structure of the Dark Triad and identify groups based on the data-driven approach. Further, a morphometric analysis was performed for the obtained groups to assess the differences in gray matter volumes between different Dark Triad profiles. We did not formulate specific hypotheses about the particular localization of possible changes in the gray matter depending on the Dark Triad indicators, but expected to detect these changes in brain structures that are associated with the ‘social brain’, primarily with the ToM network.

## 2 Materials and methods

### 2.1 Participants

A total of 129 healthy right-handed volunteers (70 women and 59 men) participated in the study. All participants were 24.4 ± 4 years old, with no history of neurological or psychological disorders and no contraindications for magnetic resonance imaging. All subjects provided written informed consent prior to the study. All procedures were conducted in accordance with the Declaration of Helsinki and were approved by the Ethics Committee of the N.P. Bechtereva Institute of the Human Brain, Russian Academy of Sciences.

### 2.2 Psychological testing and clustering procedure

The Russian version of the Dark Triad Dirty Dozen (Kornilova et al., 2015) is a 12-item self-report questionnaire of the three Dark Triad traits with the 5-point Likert scale (1 = Strongly disagree, 5 = Strongly agree) and with statements such as: “I tend to manipulate others to get my way” (Machiavellianism), “I tend to lack remorse” (psychopathy), and “I tend to want others to admire me” (narcissism). Scores for all three traits were summed and then transformed into z-scores for using in clustering procedure.

To account for the multifactorial structure of the Dark Triad and to reveal subgroups in our sample, we applied a clustering procedure using the k-means algorithm implemented in MATLAB. This method uses the squared Euclidean distance metric and the k-means++ algorithm for cluster center initialization (Arthur and Vassilvitskii, 2007). We did not use the latent profile analysis (LPA) based on structural equation modeling (SEM) results as previously described (McLarnon, 2022) primary due to the small sample size. Instead, the k-means algorithm is a simple and intuitive approach that is effective in both small (not below 50 subjects) and large data samples (Henry et al., 2015). The variance ratio criterion (VRC) was used to determine the optimal number of clusters for the current data. The maximum VRC value was obtained for *k* = 2.

### 2.3 Data acquisition and preprocessing

Magnetic resonance imaging was performed using a 3T Philips Achieva (Philips Medical Systems, Best, The Netherlands). Structural images were acquired using a T1-weighted pulse sequence (T1W-3D-FFE; repetition time [TR] = 2.5 ms; TE = 3.1 ms; 30° flip angle), recording 130 axial slices (field of view [FOV] = 240 × 240 mm; 256 × 256 scan matrix) of 0.94 mm thickness. All MRI scans were inspected for image artifacts and incidental brain abnormalities. All subjects were included in the study.

### 2.4 Voxel based morphometry analysis (VBM analysis)

The VBM analysis of structural data was performed with Statistical Parametric Mapping software (SPM12, Wellcome Department of Imaging Neuroscience, London, UK^1^) and the Computational Anatomy Toolbox 12 (CAT12^2^) running in MATLAB (MathWorks, Natick, MA). All structural data were manually reoriented to place their native-space origin at the anterior commissure. Images were corrected for magnetic field inhomogeneities and segmented into grey matter, white matter, and cerebrospinal fluid. Normalization to MNI space using the DARTEL (Diffeomorphic Anatomical Registration Through Exponentiated Lie algebra) algorithm to a 1.5 mm isotropic adult template provided by the CAT12 toolbox was performed for segmented grey matter data. Finally, the grey matter segments were smoothed with a Gaussian smoothing kernel of 8 mm. The CAT12 toolbox provides an automated quality check protocol. Therefore, quality check control for all structural data was performed to obtain so-called image quality rating (IQR) scores that were later used as an additional covariate in the statistical analysis. In addition, total intracranial volumes (TIVs) were calculated to be used as a covariate.

### 2.5 Statistical analysis of VBM data

The statistical analysis was performed for two groups of subjects. For the VBM analysis, we included the following confounders (as covariates), which can affect VBM results: sex (male/female), age, TIV (using ANCOVA), and IQR scores. The two-sample t-test was performed to test the hypothesized differences in the gray matter volume (GMV). The threshold-free cluster enhancement (TFCE) implemented in the TFCE-toolbox^3^ with 5000 permutations per test was applied. Statistical parametric maps were created based on TFCE with *p* < 0.001 on the voxel level with the family-wise error (FWE) correction for multiple comparisons. The SPM results were visualized using the MRIcron toolbox.^4^

## 3 Results

### 3.1 DDDT data clustering

**Figure 1:**
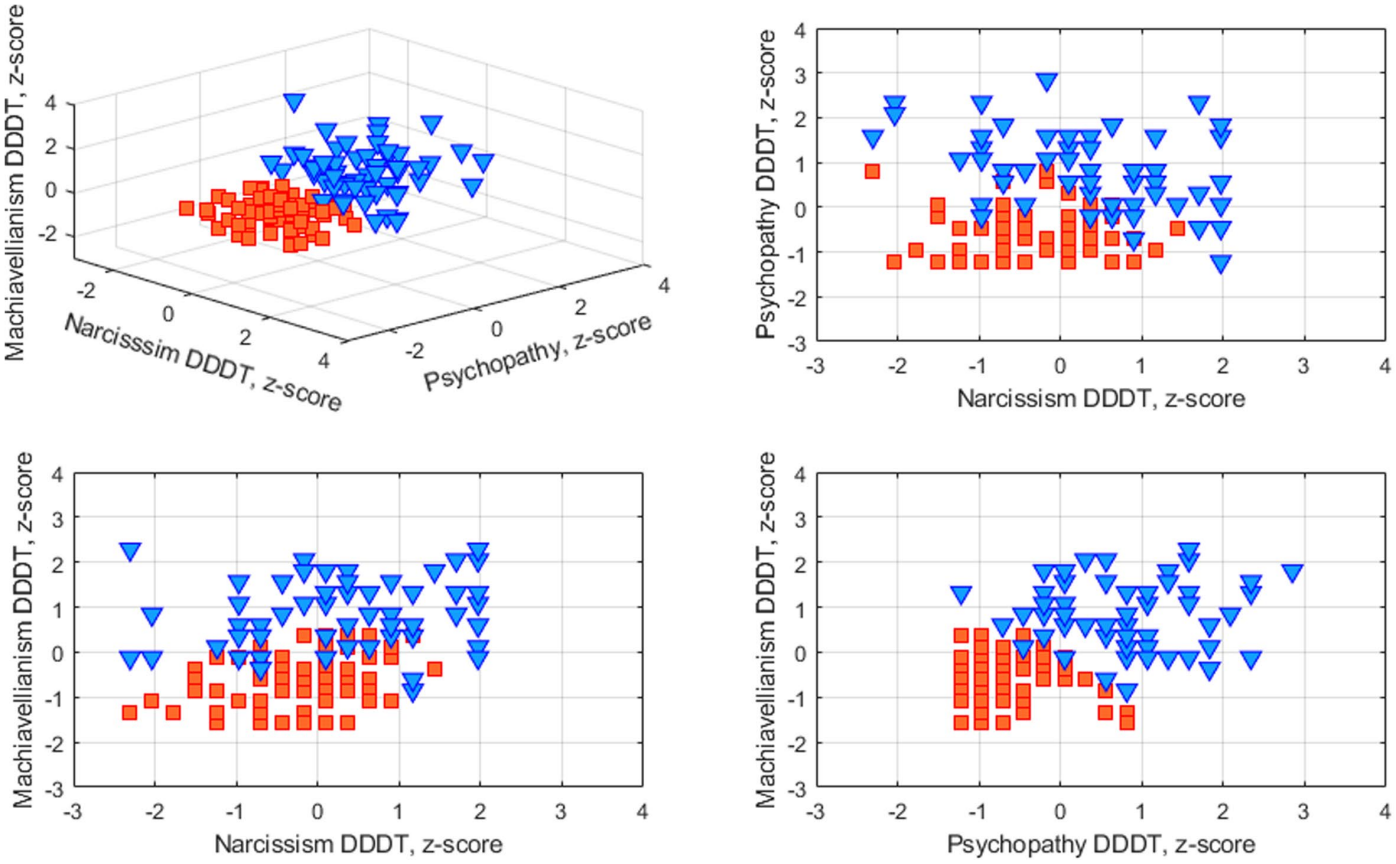
Results of DDDT data clustering using k-means algorithm.

**Figure 2:**
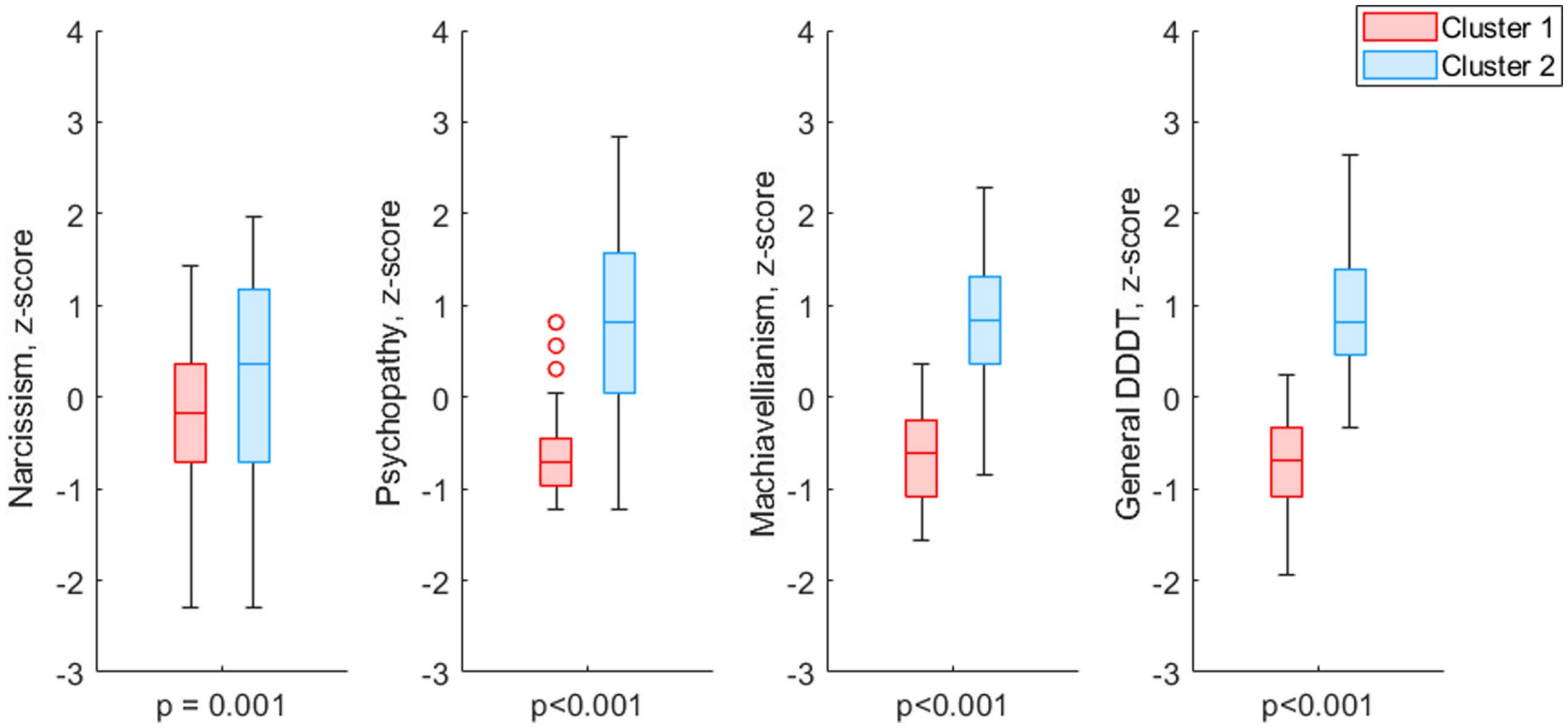
Boxplot of z-scores of DDDT subscales and general DDDT score for data clusters. Outliers are shown as red dots.

The results of data clustering are shown in Figure 1. Acquired clusters differed significantly in levels of DDDT subscales as well as in overall DDDT scores (see Table 1 and Figure 2). Clusters did not differ significantly in gender distribution (*chi-square* = 3.09, *p* = 0.08) or age (*p* = 0.26). Clusters were designated as “mid-to-high DT level” (57 subjects) and “low-to-mid DT level” (72 subjects).

**Table 1:** Demographics and scores (mean ± standard deviation) for different psychological scales in the revealed data clusters.

Psychological scale	Cluster 1 (Low-to-Mid DT Level)	Cluster 2 (Mid-to-High DT Level)	*p*-value
Age and gender			
Age	24.1 ± 3.8	24.9 ± 4.3	0.258
Gender (male/female)	28/44	31/26	0.079
Dirty dozen dark triade			
Narcissism	11.7 ± 2.9	13.8 ± 4.3	0.001
Psychopathy	6.3 ± 2.0	12.0 ± 3.4	<0.001
Machiavellianism	7.7 ± 2.3	14.0 ± 3.1	<0.001
General score	25.7 ± 4.7	39.8 ± 5.7	<0.001

DT, Dark Triad.

### 3.2 GMV differences between low-to-mid and mid-to high DT levels

**Figure 3:**
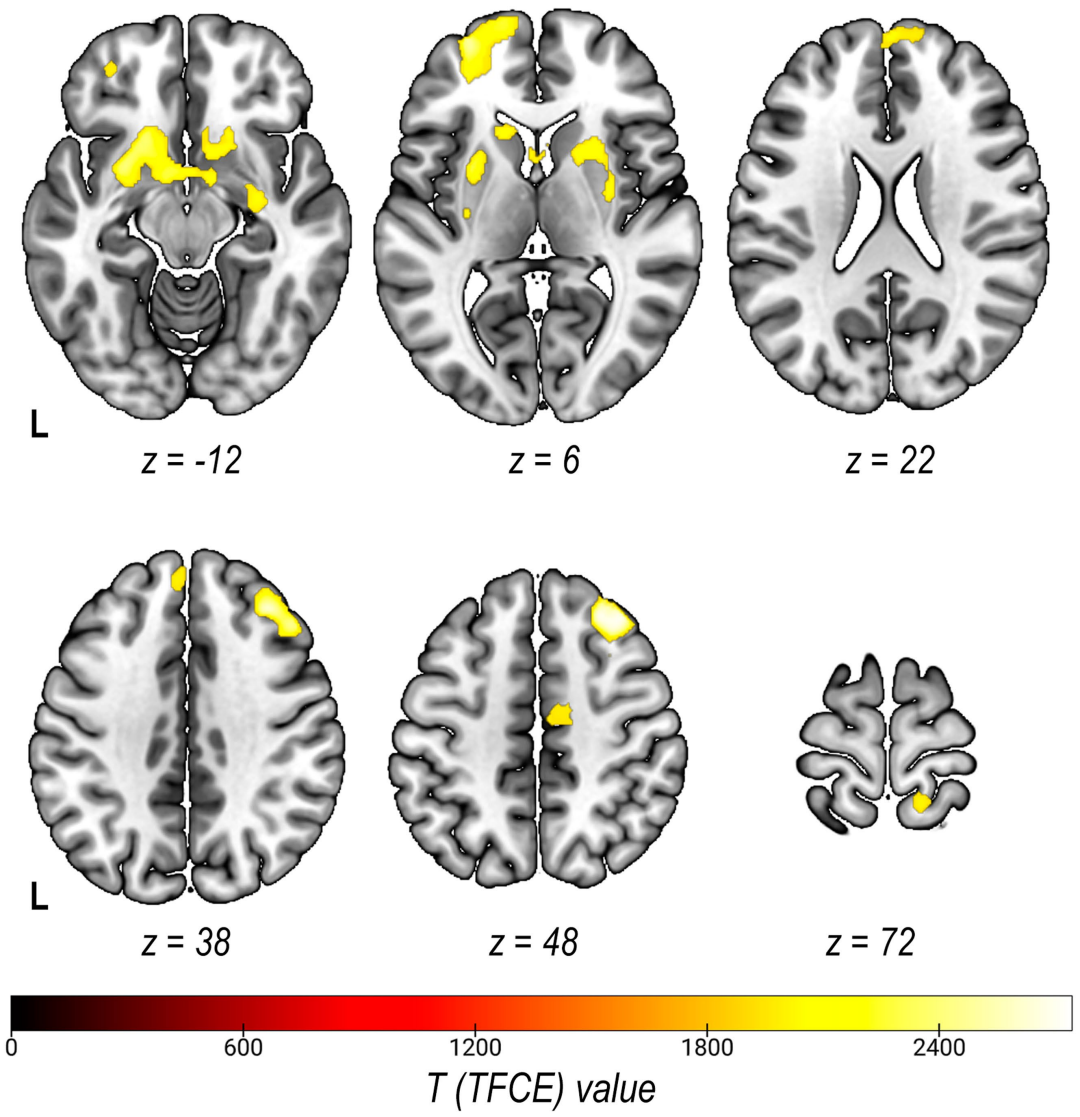
Statistical parametric maps of grey matter volume differences in subjects with Low and High DT Levels (contrast Low>High) at the TFCE voxel-level *pFWE* < 0.001, k > 80.

The VBM analysis revealed the GMV decrease in the mid-to-high DT level group in several regions including the prefrontal cortex (both medial and lateral, orbitofrontal cortex), the basal ganglia (the bilateral nucleus accumbens and putamen, the left caudate), the middle cingulate cortex as well as the right postcentral gyrus (see Table 2 and Figure 3; see also Supplementary material). Inverse contrast (High>Low) did not reveal any significant clusters.

**Table 2:** Results of VBM analysis (Low DT > High DT), TFCE voxel-level pFWE<0.001, k > 80.

No	Brain area	*k*	T (TFCE)-value	MNI coordinates		
				x	y	z
1	R MFG R SFG	1557	2744.2556	37.5	33	43.5
			2056.4529	30	10.5	57
2	R/L Accumbens R/L Putamen L Caudate R/L Septal Area	4457	2542.4211	−28.5	4.5	−7.5
			2279.8284	−7.5	3	−10.5
			2235.2615	12	21	−9
3	L MFG L SFG	1639	2497.1233	−28.5	61.5	−1.5
4	R SFG (medial)	289	2074.8376	1.5	64.5	22.5
5	R Postcentral G	121	2060.4263	15	−51	75
6	L MFG (medial)	146	2026.1814	−1.5	51	40.5
7	R MCC/PCC	102	2024.5991	10.5	−9	49.5
8	L Anterior OFC	97	2009.1361	−33	48	−18

MFG, middle frontal gyrus; SFG, superior frontal gyrus; MCC/PCC, middle/posterior cingulate cortex.

## 4 Discussion

With this study, we aimed at assessing the impact Dark Triad profiles have on anatomical features of the brain. To define the profiles, we used a short DDDT questionnaire – a psychological tool for rapid integral assessment of the Dark Triad, consisting of 12 questions, with four questions per each of the Dark Triad traits – narcissism, psychopathy, Machiavellianism. All of these dark personality traits are complex constructs, and for each of these traits individual questionnaires determine a multifactorial structure (MACH-IV, NPI, SRP). Thus, according to the MACH-IV questionnaire, the following factors can be identified in the structure of Macchiavelinism: positive and negative interpersonal tactics, a cynical view of human nature, and disregard for moral norms (Muris et al., 2017). The multifactorial structure of narcissism includes lust for power, a sense of superiority, exhibitionism, feeling entitled, vanity, tendencies to exploit other people’s resources, and self-confidence (del Rosario and White, 2005). Finally, the structure of psychopathy according to the SRP questionnaire includes interpersonal manipulation, callousness, erratic lifestyle, and a tendency toward criminal behavior (Massa and Eckhardt, 2017). A comparison of the DDDT questionnaire and individual tests for assessing dark personality traits showed that the DDDT allows evaluating only three factors of narcissism out of seven (entitlement, superiority, exhibitionism), two factors of Machiavellianism (manipulative tactics, disregard for moral norms) and one factor of psychopathy (callousness) (Muris et al., 2017). We find it important for understanding the psychological differences identified for the clustering groups.

While the vast majority of research articles on neuroanatomic correlates of Dark Triad were focused only on narcissism, psychopathy and machiavellianism separately, our data-driven clustering approach provided for a more integrative assessment. To isolate data-driven profiles using psychometric indicators of the Dark Dozen questionnaire, an algorithm for clustering data using k-means was applied. As a result, data were divided into two groups based on the clustering effectiveness evaluation. The further analysis showed that the obtained groups significantly differed in terms of each of the Dark Triad subscales, as well as its overall cumulative score according to the DDDT questionnaire. At the same time, the groups did not differ significantly in gender distribution and age. Hence, these results demonstrate the effectiveness of the chosen clustering method (k-means) in respect to our data. These groups were used in the further morphometric analysis that revealed a gray matter volume decrease in individuals with prominent dark personality traits in a number of structures, including the medial and dorsolateral prefrontal, the orbitofrontal cortex, subcortical nuclei (the nucleus accumbens and left shell), the middle cingulate cortex, and the right precentral gyrus.

The results of the morphometric analysis confirmed our assumption about differences between dark trait prominence levels manifested in the volume of structures related to the processing of socially significant information. We detected a cluster in the area of the medial prefrontal cortex that overlaps the ToM system area. One of the possible ways to describe the ToM system is to define cognitive and affective domains. The affective domain is usually related to understanding of the emotional states of others, while the cognitive domain is assumed to be involved in understanding thoughts and intentions (Molenberghs et al., 2016). We registered a decrease in gray matter volumes only for the medial prefrontal cortex (mPFC), an element of the affective domain. The involvement of the mPFC in the affective ToM processes has been shown in many studies (see for review Lieberman et al., 2019). More specifically, patients with damage to the ventral mPFC regions performed worse on the task of recognizing mental states than control groups – the task that should involve the affective ToM (Shamay-Tsoory et al., 2006; Shamay-Tsoory and Aharon-Peretz, 2007; Leopold et al., 2012). A decrease in the medial prefrontal cortex gray matter volume was associated with high levels of psychopathy (de Oliveira-Souza et al., 2008; Yang and Raine, 2009; Yang et al., 2010; Lam et al., 2017), although in some studies the dependence was reversed (Korponay et al., 2017).

A gray matter volume decrease in individuals with higher DT prominence was also detected in the right dorsolateral prefrontal cortex (dlPFC), one of the central executive network nodes that is also involved in processing of emotions and emotional stimuli. In particular, according to the term-based meta-analysis of Neurosynth, one of the terms associated with the cluster obtained in our study is reappraisal associated with the emotion regulation. Cognitive reassessment is one of the regulatory mechanisms during which a person re-evaluates a situation and its significance in order to change its emotional impact (Gross, 2015). It has been shown that psychopathy is characterized by low reliance on this mechanism, while for Machiavellianism and narcissism, this dependence has not been previously identified (Walker et al., 2022). The ventromedial and the dorsolateral prefrontal cortex, as previously shown, play different roles in emotion processing: for example, the dlPFC is involved in the control and regulation of emotional experience valence, while the ventromedial prefrontal cortex (vmPFC) may be involved in the suppression of arousal caused by emotional stimuli (Nejati et al., 2021). In addition, structural post-traumatic changes in the left dlPFC were associated with a high level of Machiavellianism according to the TDM-IV scale, in particular, with a high level on the “Machiavellian Views” scale (Cohen-Zimerman et al., 2017).

In addition, our study revealed a gray matter volume decrease in the left orbitofrontal cortex (OFC). There is evidence of the OFC involvement in processes associated with both affective and cognitive empathy (Brink et al., 2011). It is believed that the OFC may be involved in the modulation of empathy by various factors, such as social distance or gender (Hillis, 2014). In addition, the OFC is reportedly involved in emotional processing: a decrease in the OFC gray matter volume negatively correlated with the degree of emotional dysregulation (Petrovic et al., 2016). An OFC gray matter volume decrease was also associated with decreased ability to track dynamically changing emotions (Goodkind et al., 2012). Finally, according to a recent meta-analysis of morphometric studies, the gray matter volume in the orbitofrontal and prefrontal cortex negatively correlated with the degree of impulsivity (Pan et al., 2021) inherent in dark personality traits – psychopathy and to a lesser extent narcissism (Jones and Paulhus, 2011; Ball et al., 2018; Malesza and Kalinowski, 2021).

Summarizing all the above, the results of our study demonstrate a possible relationship between the prominence of the Dark Triad traits and the volume of structures associated with socio-emotional functions, such as empathy and emotional regulation. The neuroanatomic data agree with the outcomes of psychological works. Namely, a number of studies and meta-analyses have demonstrated a negative correlation between various components of emotional intelligence and the Dark Triad traits, in particular, Machiavellianism and psychopathy, although no correlation has been shown for narcissism (Miao et al., 2019; Michels and Schulze, 2021). At the same time, we observed no changes in the gray matter volume in structures related to sociocognitive functions, for example, the cognitive ToM domain. Manipulative behavior characterizes the Dark Triad and manifests itself in anatomical and functional features of the cognitive ToM domain structures.

Applying the morphometric analysis, we revealed an extensive cluster of gray matter volume reduction in individuals with a higher DT level in the structures of the mesolimbic system – the nucleus accumbens, the ventral striatum, the septal region – often referred to collectively as the Basal Forebrain (BF). These structures are anatomical correlates of reward processing and behavior regulation. Moreover, Hoffman and O’Connell attribute the above-mentioned areas to the social decision-making network in mammals (O’Connell and Hofmann, 2011). Additionally, Morelli and colleagues link activations in the septal region with variants of empathy (to pain, anxiety, happiness level) that predicted daily help to other people (Morelli et al., 2014). Likewise, numerous works indicate the BF role in altruistic behavior and charitable donations (Moll et al., 2006; Harbaugh et al., 2007; Kirk et al., 2016) and expectation of reward (Schultz et al., 1997). Many pathological conditions in which violations of various aspects of social behavior are observed (ASD, FTLD, etc.) are accompanied by structural and functional changes in the BF (Nickl-Jockschat et al., 2011; Riva et al., 2011; Convery et al., 2020; Schulz et al., 2023). Since a violation of social interactions is also observed in individuals with a higher DT level (see Introduction), the neuroanatomic basis of such antisocial behavior may be a lower volume of BF structures.

The present study has a limitation that is related to the psychological assessment technique we used for the Dark Triad. The DDDT, as mentioned above, while being a widely used simple and reliable instrument, does not fully cover the multifactorial structure of the individual traits of the Dark Triad as well as does not count associations between prominence of Dark Triad traits and both empathy (Pajevic et al., 2018; Heym et al., 2019) and aggression (Pailing et al., 2014; Jones and Neria, 2015). Potential solutions to this limitation for future research are (1) the use of the SD3 questionnaire, which is more inclusive of the multifactor structure of the Dark Triad, (2) the use of separate questionnaires for each of the Dark traits and (3) the use of separate questionnaires for associated traits like aggression and empathy. In addition, the use of structural equation modeling of psychological data on larger sample sizes would result in additional insight about the structure of the Dark Triad profile with the identification of their neuroanatomical correlates.

## 5 Conclusion

One way toward clarifying the nature of the ‘social brain’ is to describe the complex relations between psychometric indicators of dark personality traits. Our study helped identify neuroanatomic correlates of Dark Triad trait prominence levels via clustering data from the DDDT questionnaire. We were able to elucidate the neurobiological basis of social behavior in individuals with a higher DT level by analyzing gray matter volume variance in brain areas that provide for different aspects of social interactions. The volume decrease in structures associated with emotional regulation (OFC, vmPFC/dlPFC), empathy (OFC, MCC), and the reward system (basal forebrain) complements assumptions about changes in the operation of these systems in individuals with various Dark Triad trait prominence levels.

## Data availability statement

The raw data supporting the conclusions of this article will be made available by the authors, without undue reservation.

## Ethics statement

The studies involving humans were approved by Ethics Committee of the N.P. Bechtereva Institute of the Human Brain, St. Petersburg, Russia. The studies were conducted in accordance with the local legislation and institutional requirements. The participants provided their written informed consent to participate in this study.

## Author contributions

AM: Data curation, Formal analysis, Investigation, Methodology, Validation, Visualization, Writing – original draft, Writing – review & editing. AK: Conceptualization, Investigation, Methodology, Supervision, Validation, Writing – original draft, Writing – review & editing. MZ: Formal analysis, Investigation, Writing – original draft. VK: Formal analysis, Visualization, Writing – original draft. RM: Data curation, Formal analysis, Investigation, Methodology, Software, Writing – original draft. KB: Formal analysis, Writing – review & editing. OY: Data curation, Methodology, Writing – review & editing. MV: Data curation, Investigation, Validation, Writing – review & editing. DC: Funding acquisition, Project administration, Resources, Writing – review & editing. MD: Funding acquisition, Resources, Supervision, Writing – review & editing. MK: Conceptualization, Data curation, Methodology, Project administration, Supervision, Validation, Writing – original draft, Writing – review & editing.

## Footnotes

^1^ www.fl.ion.ucl.ac.uk/spm

^2^ http://dbm.neuro.uni-jena.de/cat.html

^3^ http://dbm.neuro.uni-jena.de/tfce

^4^ https://www.nitrc.org/projects/mricron
