# Supplementary material for: Dark triad personality traits are associated with decreased grey matter volumes in ‘social brain’ structures
Source: Front Psychol. 2024 Jan 12;14:1326946. doi: 10.3389/fpsyg.2023.1326946 (PMC10811166; doi:10.3389/fpsyg.2023.1326946)
Supplement: Supplementary file 1 [file Data_Sheet_1.docx]

**Supplementary Materials**

**Figure 1. Comparison of VBM results at TFCE voxel level pFWE<0.001 and amygdala location using Harvard-Oxford parcellation.**

**
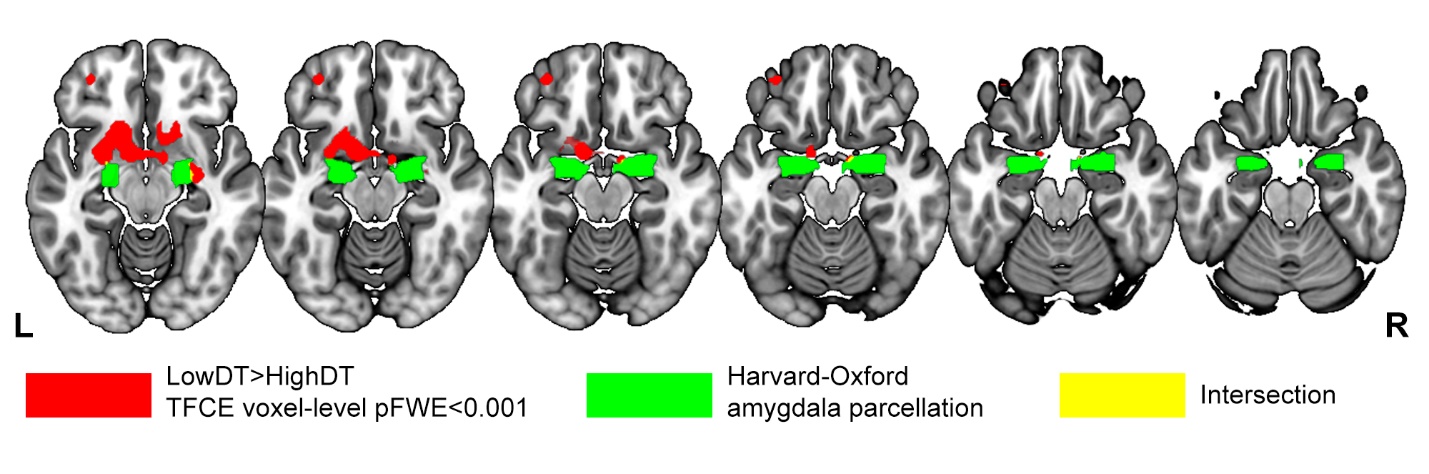
**

**Figure 2. Comparison of VBM results at TFCE voxel level pFWE<0.05 and amygdala location using Harvard-Oxford parcellation.**

**
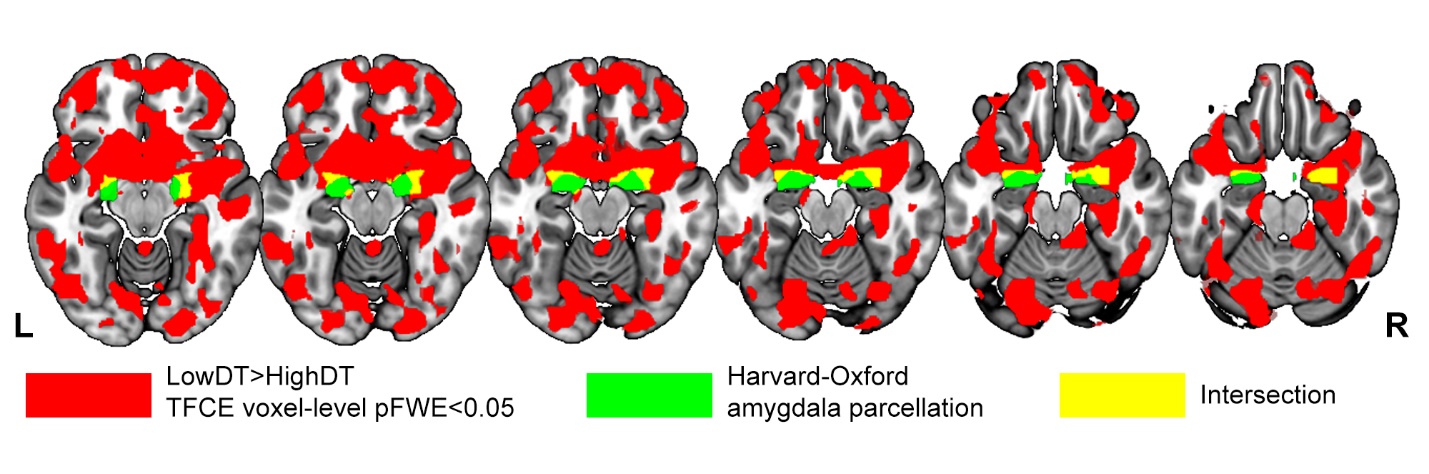
**

**Figure 3. Results of VBM analysis (Low DT > High DT) using empathy (IRI) and aggression (BPAQ) measures as covariates, TFCE voxel-level pFWE<0.001, k > 80.**

**
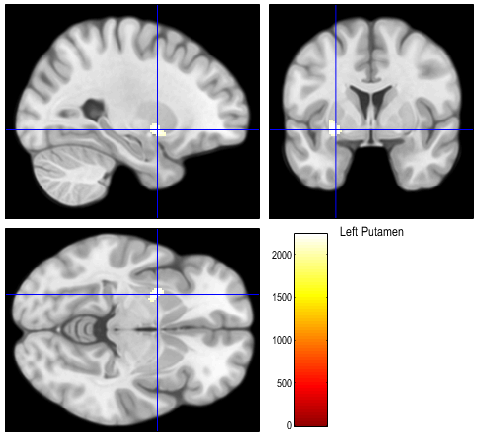
**

**Figure 4. Results of VBM analysis (Low DT > High DT) using empathy (IRI) and aggression (BPAQ) measures as covariates, TFCE voxel-level pFWE<0.01, k > 80.**


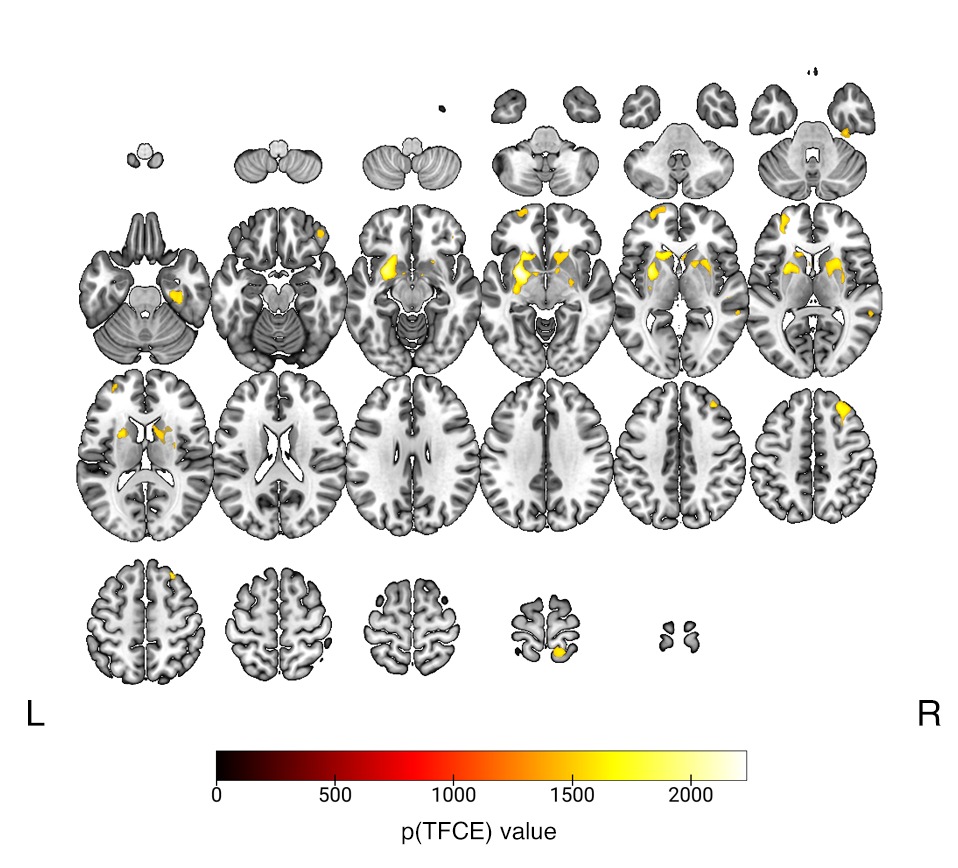


**Figure 5. Comparison of VBM findings for analysis with/without control for empathy/aggression (total scores for both IRI and BPAQ).**

**
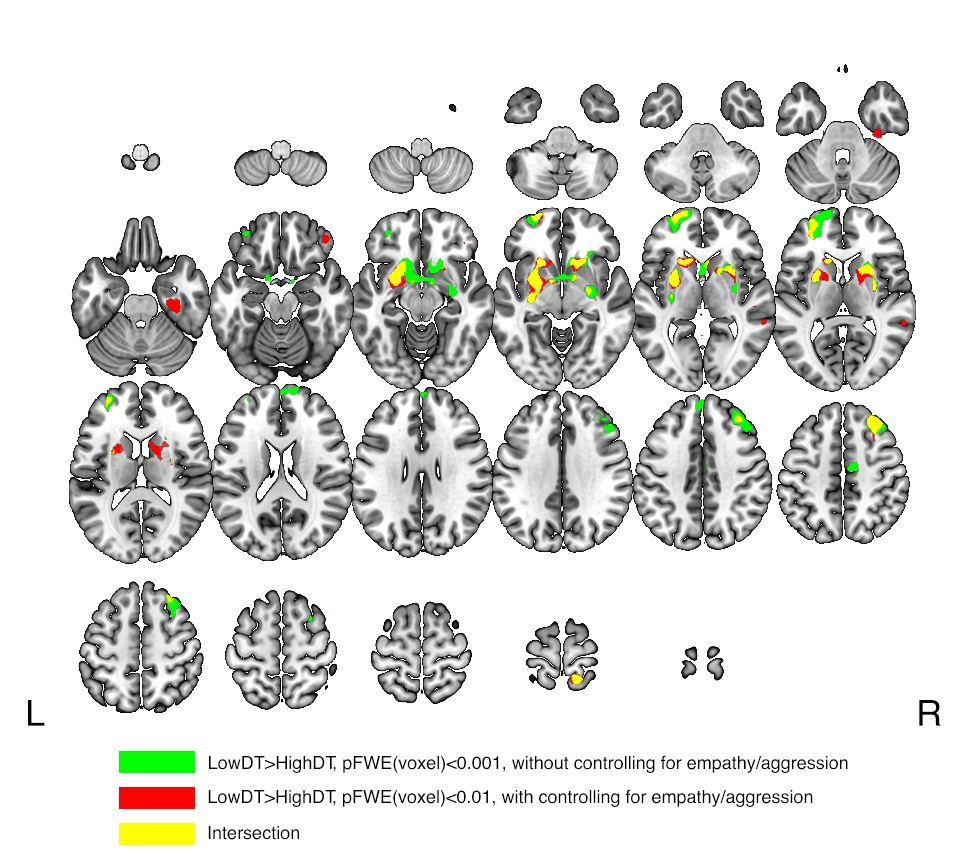
**
